# Testosterone replacement in young male cancer survivors (TRYMS) - pragmatic adaptation of trial design for a trial struggling with recruitment

**DOI:** 10.1186/1745-6215-16-S2-O79

**Published:** 2015-11-16

**Authors:** Isabelle Smith, Jayne Swain, Helen Marshall, Alexandra Smith, Jennifer Walsh, Richard Ross, Sarah Brown

**Affiliations:** 1 Leeds Institute of Clinical Trials Research, Leeds, UK; 2 University of Sheffield, Sheffield, UK

## 

TRYMS is a multicentre, randomised, double-blinded, parallel group, placebo controlled phase IV trial in young male cancer survivors with low testosterone levels. The trial is designed to investigate whether testosterone treatment for 26 weeks will result in a reduction of truncal fat mass and increase in participant-reported physical functioning scores.

The general consensus worldwide is that testosterone levels ≤12nmol/L are deemed to be low; however a previous study of 200 healthy volunteers found that testosterone≤10nmol/L would be deemed to be low. The trial team considered only including patients with testosterone≤10nmol/L but acknowledged this would leave an evidence gap for patients with testosterone between 10-12nmol/L. Therefore TRYMS was designed to determine whether testosterone replacement is effective in men with low testosterone and with borderline low testosterone by powering the study to look at each subgroup individually. A sample size of 268 participants was required after accounting for imbalanced recruitment to the subgroups.

However, opening TRYMS took longer than anticipated and recruitment was substantially slower than expected; the trial design was adapted to combine the subgroups for primary analysis, reducing the minimum sample size to 112 participants. The trial was granted a recruitment extension and continued to recruit beyond the minimum sample size to maximise precision of the treatment effect estimates in each subgroup; this analysis is exploratory but still considered useful to inform clinical practice. This adaption of the trial design was a pragmatic approach for a trial struggling with recruitment, whilst still able to provide clinically useful results.

